# *Myrciaria jaboticaba* Fruit Peel: Bioactive Composition as Determined by Distinct Harvest Seasons and In Vitro Anti-Cancer Activity

**DOI:** 10.3390/plants13202907

**Published:** 2024-10-17

**Authors:** Roberto de Paula do Nascimento, Julia Soto Rizzato, Gabriele Polezi, Hatim Boughanem, Non Gwenllian Williams, Renata Galhardo Borguini, Manuela Cristina Pessanha de Araujo Santiago, Mario Roberto Marostica Junior, Lee Parry

**Affiliations:** 1 Laboratório de Nutrição e Metabolismo, Departamento de Ciência de Alimentos e Nutrição, Faculdade de Engenharia de Alimentos, Universidade Estadual de Campinas, Campinas 13083-862, SP, Brazil; jsotorizzato@gmail.com (J.S.R.); gpolezi@gmail.com (G.P.); mmarosti@unicamp.br (M.R.M.J.); 2 Prevention and Early Detection Laboratory, European Cancer Stem Cell Research Institute, School of Biosciences, Cardiff University, Cardiff CF24 4HQ, UK; h.b.boughanem@gmail.com (H.B.); williamsng1@cardiff.ac.uk (N.G.W.); parryl3@cardiff.ac.uk (L.P.); 3 Department of Endocrinology and Nutrition, Virgen de la Victoria University Hospital, 29010 Malaga, Spain; 4 Unidad de Gestión Clinica Medicina Interna, Lipids and Atherosclerosis Unit, Maimonides Institute for Biomedical Research in Córdoba (IMIBIC), Reina Sofia University Hospital, University of Córdoba, 14004 Córdoba, Spain; 5 Spanish Biomedical Research Center in Physiopathology of Obesity and Nutrition (CIBERObn), Instituto de Salud Carlos III, 28029 Madrid, Spain; 6 Empresa Brasileira de Pesquisa Agropecuária, Embrapa Agroindustria de Alimentos, Rio de Janeiro 23020-470, RJ, Brazil; renata.borguini@embrapa.br (R.G.B.); manuela.santiago@embrapa.br (M.C.P.d.A.S.)

**Keywords:** agro-industrial byproduct, anthocyanins, anti-colony-forming activity, anti-proliferative action, Caco-2 cells, colorectal cancer, cyanidin-3-*O*-glucoside, delphinidin-3-*O*-glucoside, gallic acid, phenolic compounds

## Abstract

Jaboticaba (*Myrciaria jaboticaba*) is a recognizable and unique crop from Brazil. The fruit’s byproducts are currently being studied, given their bioactive composition and promising anti-cancer potential. It is not evident, however, if different harvesting seasons can modify the chemical profile and antioxidant capacity of jaboticaba fruit fractions. Furthermore, as there is limited data for jaboticaba’s anti-proliferative effects, additional assessments are required to improve the robustness of these findings. Therefore, this study aimed to determine the composition of the peel of jaboticaba collected in two periods (May—off-season, sample 1—and August–October—peak season, sample 2) and test the peel’s richest anthocyanin sample against colorectal cancer (CRC) cell lines. To accomplish this, proximate, spectrophotometric, and chromatographic analyses were performed in two freeze-dried samples; and anti-proliferative and/or colony-forming assays were carried out in Caco-2, HT29, and HT29-MTX cells. As a result, sample 2 showed the highest levels of polyphenols overall, including flavonoids and anthocyanins. This sample displayed significative higher contents of cyanidin-3-*O*-glucoside (48%) and delphinidin-3-*O*-glucoside (105%), in addition to a superior antioxidant capacity (23% higher). Sample 1 showed higher amounts of total protein, gallic acid (20% higher), and specific carotenoids. An aqueous extract from sample 2 was tested against CRC, showing anti-proliferative effects for Caco-2 cells at 1 and 2 mg/mL concentrations, with IC50 values of 1.2–1.3 mg/mL. Additionally, the extract was able to inhibit cell colony formation when tested at both low and high concentrations. In conclusion, jaboticaba collected in the main season stands out regarding its polyphenol composition and holds potential against cancer cell growth.

## 1. Introduction

Brazil contains a diversity of native plants throughout its territory [1]. Biomes such as the Amazon, Atlantic Forest, and Cerrado are home to non-conventional tree exemplars, which provide tasteful and nutritious fruits of enormous industrial and commercial potential [2]. In recent years, a few indigenous samples have been studied regarding their bioactive composition and potential health effects. Jaboticaba (*Myrciaria* spp.), from the Atlantic Forest, stands out as one the most promising crops of Brazil [3,4,5]. The tree provides characteristic red-to-purple globose berries of sweet and appreciable pulp. Among jaboticaba fractions, however, the peel and seed have shown the highest bioactive potential [6]. These byproducts are regarded as rich sources of dietary fibers and polyphenols [7]. Most commonly, studies have suggested that jaboticaba peel possesses a high pro-health potential given its abundance in anthocyanins, phenolic acids, and tannins, in addition to a high antioxidant capacity [8]. The jaboticaba fruit is largely harvested for consumption or commercial purposes in the year’s second semester, mostly during August–November [9], with collection at other times being less common. Studies addressing possible composition differences between jaboticaba’s harvest seasons are non-existent and may be important to reveal the fruit’s other chemical qualities, in addition to its best industrial and pharmacological utilization.

In preclinical studies, jaboticaba’s powder and extracts have shown the capacity to inhibit the proliferation of breast, colon, and lung cancer cells in vitro, in addition to preventing colorectal cancer (CRC) in animals [10,11,12]. A recent study showed the positive effects of jaboticaba’s pectin on CRC cells in vitro. The treatment with purified pectin was able to reduce the proliferation of HCT116 and HT29 cells, as well as to inhibit galectin-3, a protein associated with aggressive types of cancer [13]. In another study, diet-enriched jaboticaba peel powder was capable of inhibiting intestinal inflammation, consequently preventing any formation of carcinoma [14]. Other investigations have suggested the benefits of jaboticaba’s anthocyanins on CRC by testing various extracts in both in vitro and animal models. While the in vitro studies demonstrated that the extracts of jaboticaba peel can reduce the proliferation of HT29 monolayers and spheroids [15,16], the ones using animals showed that anthocyanin-rich preparations from the whole fruit or seed can suppress the formation of aberrant crypts and modulate the intestinal microbiota of rats, respectively [17,18]. Despite a few investigations having indicated the anti-CRC potential of jaboticaba peel, the evidence is still not robust enough to list the fruit as a new therapeutic candidate. Since conventional medicine can sometimes be ineffective and offer resistance [19], the continuing search for complementary ways of handling CRC may eventually help improve health care. Disease initiation blockage by natural products could have an enormous impact on health expenditure, considering that up to 90% of CRC cases are said to be preventable [20].

All things considered, our study had as main objectives to (a) understand the differences in chemical composition and antioxidant capacity between the peel of jaboticaba collected in two distinct harvest periods (peak season and off-season), and (b) investigate the action of the richest anthocyanin sample against CRC cell lines (Caco-2, HT29, and HT29-MTX).

## 2. Material and Methods

### 2.1. Chemicals

The MTT assay kit (cell proliferation) was purchased from Abcam (Cambridge, UK). 2,2′-Azobis(2-amidinopropane) dihydrochloride was purchased from Cayman Chemical (Ann Arbor, MI, USA). Pelargonidin-3-*O*-glucoside (P3G) standard (CAS 18466-51-8), with ≥95% purity, was purchased from PhytoLab (Vestenbergsgreuth, Germany). 4-Hydroxybenzoic acid (CAS 99-96-7), catechin (CAS 154-23-4), cyanidin-3-*O*-glucoside (C3G) or kuromanin chloride (CAS 7084-24-4), delphinidin-3-*O*-glucoside (D3G) (CAS 6906-38-3), ellagic acid (CAS 476-66-4), epicatechin (CAS 490-46-0), ferulic acid (CAS 537-98-4), gallic acid (CAS 149-91-7), *p*-coumaric acid (CAS 501-98-4), protocatechuic acid (CAS 99-50-3), quercetin (CAS 117-39-5), rutin (CAS 153-18-4), and syringic acid (CAS 530-57-4) standards, all with ≥95% purity, were purchased from Sigma Aldrich (St. Louis, MO, USA). The analytical standards for carotenoids were previously isolated in the laboratory (purity grade ≥ 95%), according to Kimura and Rodriguez-Amaya [21]. 2,4,6-Tripyridyl-S-triazine, 6-hydroxy-2,5,7,8-tetramethylchroman-2-carboxylic acid or Trolox, bovine serum albumin, and Folin–Ciocalteu reagent were all purchased from Sigma Aldrich (St. Louis, MO, USA). Fluorescein was purchased from Synth (Diadema, SP, Brazil). Acetone, acetonitrile, methanol, methyl tert-butyl ether, and petroleum ether, all high-performance liquid chromatography grade, were all purchased from Tedia (Rio de Janeiro, RJ, Brazil). Hydrochloric acid (37%) and phosphoric acid (85%) were purchased from Tedia (Rio de Janeiro, RJ, Brazil). Dulbecco’s Modified Eagle Medium (DMEM), penicillin 5 units/mL and streptomycin 5 μg/mL, phosphate-buffered saline (PBS) pH 7.4, and trypsin-EDTA 0.25% were all purchased from Thermo Fisher (Paisley, UK).

### 2.2. Fruit Registry, Collection, and Processing

As jaboticaba is a native Brazilian crop, the local and overseas studies were registered at *SisGen/Brazil*, protocol numbers AD872CA and RA12F13, respectively. The fruit from the *Myrciaria jaboticaba* (Vell.) O. Berg tree, jaboticaba Sabará, was obtained during the year 2019 in two distinct harvest periods, these being May (autumn, off-season)—sample 1—and August–October (winter/spring, peak season)—sample 2. The samples were collected at Casa Branca, São Paulo state, Brazil, coordinates 21°53′42.1″ S 47°02′10.9″ W. The fruits were transported to the school facilities and stored at −20 °C. Afterward, they were thawed and properly sanitized using 1% sodium hydrochloride in tap water (*v*/*v*). Only the fruit peel was used for the experiments. The peel was manually separated and frozen at −20 °C for freeze-drying. The freeze-drying conditions followed the equipment and conditions described by Leite-Legatti et al. [22]. Briefly, once frozen, the peel samples were dried in equipment (LP1010, Liobras, São Carlos, SP, Brazil) programmed at 30 °C and 300 μmHg for 95 h [22]. After lyophilization, the materials were ground in a mill to a powder, being subsequently stored at −20 °C for further analyses.

### 2.3. Proximate and Spectrophotometric Analyses

Proximate analyses were performed in the freeze-dried samples, including moisture, ashes [23], lipids [24], and protein [25]. The total carbohydrate content was estimated based on the sum of moisture, ashes, lipids, and protein. For the spectrophotometric analyses, the samples were extracted according to Nascimento et al. [26]. Initially, the powders were mixed with a hydroalcoholic solution composed of 46% ethanol in distilled water, with pH 1 adjusted with hydrochloric acid. The sample-to-solvent ratio used was 1:20. The mixture was then subjected to an ultrasound bath for 10 min, with the water temperature at 30 °C. After 10 min, filtration was performed using a 15 cm diameter paper filter. The procedure was repeated three times with the residues of the filtration in order to extract more compounds from the samples [26]. The extracts were produced in triplicate and kept at −20 °C. Afterward, the following analyses were carried out: total phenolic content (TPC) by Folin–Ciocalteu (gallic acid curve: 16–100 µg/mL) [27], total flavonoids (catechin curve: 8–192 µg/mL) [28], monomeric anthocyanins by the pH differential method [29], and antioxidant capacity assays ferric-reducing antioxidant power (FRAP) [30] and oxygen radical absorbance capacity (ORAC) (Trolox curves) [31]. A microplate spectrophotometer coupled with Gen5™ 2.0 data analysis software was used for the analyses. Additionally, an analysis of total carotenoids was performed by ultraviolet–visible spectroscopy, according to the extraction and analytic procedures by Rodriguez-Amaya [32]. The analyses were carried out in duplicate or triplicate. Except for moisture, all proximate, spectrophotometric, and antioxidant capacity results were demonstrated as dry weight of jaboticaba peel powder.

### 2.4. Chromatographic Analyses

The samples were also analyzed by high-performance liquid chromatography coupled with a diode array detector (HPLC-DAD). The chromatographic analyses were carried out in a Waters^TM^ Alliance model 2690/5 coupled with a Waters^TM^ DAD model 2996, using Empower^TM^ software version 1. Ultrapure water (0.054 µS/cm) from the Millipore^®^ Milli-Q system (Milford, MA, USA) was used throughout the analyses. Anthocyanins, other flavonoids and phenolic acids, and carotenoids were extracted and quantified following the studies of Gouvêa et al. [33], Nascimento et al. [34], and Rodriguez-Amaya [32] and Pacheco et al. [35], respectively. All procedures were carried out in triplicate.

For anthocyanins, the freeze-dried samples were mixed with a 10% formic acid solution in methanol (sample-to-solvent ratio of 1:20) with stirring for 1 min, followed by a 10 min ultrasound extraction, and centrifugation at 6000 rpm for 10 min. The extraction process was repeated until solution discoloration. The volume obtained was completed to 10 mL by adding more 10% formic acid in methanol. About 1 mL of the solution was microcentrifuged at 12,000 rpm for 3 min. The solvent was evaporated under nitrogen flow until dry and then resuspended with the same amount (1 mL) of 10% formic acid in methanol. The extract was transferred to a vial and taken to the chromatograph injector. The following were the conditions of the chromatograph: Thermo Scientific C18 BDS (100 mm × 4.6 mm; 2.4 μm) column, column temperature at 40 °C, flow of 1.0 mL/minute, 20 µL injection. The elution mode gradient used 5% formic acid (phase A) and acetonitrile (phase B), starting with 95% of phase A and 5% of phase B; 15 min 87% A and 13% B; 16.5 min 86% A and 14% B; 18 min 95% A and 5% B, with a run time of 20 min [33]. The compounds were identified by comparing their retention time and ultraviolet/visible absorption spectra with those of the analytical standards. The quantification of anthocyanins (C3G, D3G, and P3G) was performed by external standardization.

For other flavonoids and phenolic acids, the samples were extracted with methanol–water (50:50, pH 2) (sample-to-solvent ratio of 1:20) followed by mechanical stirring for 1 h and centrifugation (5000× *g*) for 10 min. A solution of acetone–water (70:30, *v*/*v*) was added to the residue, and the mechanical stirring and centrifugation steps were repeated. About 3 mL of the two supernatants were mixed and transferred to a 1.5 mL vial for the chromatographic injection. Additionally, the solid sample residues were submitted to the extraction of hydrolyzed phenolic compounds. Alkaline hydrolysis was continued with a 5 mL solution of 2 M Sodium hydroxide containing 1% ascorbic acid and 10 mM EDTA. This solution was added to the samples, followed by heating at 61–63 °C for 60 min, and immediately after this 1.5 mL of 6 M hydrochloric acid was added for the acid hydrolysis. This solution was vortexed for 10 s, left to cool to room temperature, and then centrifuged (2700 rpm) for 10 min. The supernatant was collected and 6.5 mL of ethyl acetate was added. The organic phase was separated and the extraction with ethyl acetate was repeated. The organic fraction was dried under a nitrogen gas flow and then diluted in methanol for the chromatographic analysis. The following were the conditions of the chromatograph: Thermo Hypersil BDS C_18_ column (100 × 4.6 mm × 2.4 µm), mobile phase flow at 1 mL/minute, injection volume of 10 µL, run time of 28 min, and an elution mode gradient composed of an aqueous solution of 0.15% phosphoric acid (95%) and acetonitrile (5%) [34]. The compounds were identified by comparing their retention time and the ultraviolet–visible absorption spectra with those of the analytical standards. The quantifications of other flavonoids (catechin, epicatechin, quercetin, and rutin) and phenolic acids (4-hydroxybenzoic acid, ellagic acid, ferulic acid, *p*-coumaric acid, protocatechuic acid, and syringic acid) were performed by external standardization.

Finally, for carotenoids, the extraction was conducted with approximately 0.2 g of the samples, which were macerated with celite and acetone. The extraction procedure was repeated until the sample no longer exhibited the characteristic color of carotenoids. The acetone extract was transferred quantitatively to a separator funnel containing petroleum ether and washed, at least five times, with ultrapure water. The ether extract was filtered through anhydrous sodium sulfate, collected in 100 mL volumetric flasks, and completed with petroleum ether. A 5 mL aliquot was separated for a further saponification reaction with 5 mL of potassium hydroxide solution 10% in methanol (10:90, *v*/*v*) at room temperature and a reaction time of 16 h [32]. The carotenoid profile was determined by HPLC as described by Pacheco et al. [35], using a C30 column (S-3 Carotenoid, 4.6 mm × 250 mm, YMC™), a gradient elution of methanol, and methyl terc-butyl ether, with DAD. The flow rate was 0.8 mL/minute and the run time was 28 min. The compounds were identified by comparing their retention time and ultraviolet–visible absorption spectra with those of the analytical standards. The quantification of carotenoids (α-carotene, β-carotene, β-cryptoxanthin, and lutein) was performed by external standardization.

All chromatographic results were demonstrated as dry weight of jaboticaba peel powder.

### 2.5. Anti-Proliferative and Colony Formation Assays

An aqueous extract of the richest anthocyanin sample was prepared for use in the anti-cancer assays. We decided to use an aqueous extract for the health-related studies. This represents an easy and practical preparation, in addition to being a more green/less toxic option, but still highly functional, which could be well translated for human handling and consumption. Moreover, this aqueous extract has shown positive effects in studies conducted by our research group through pre-clinical models of chronic diseases [36,37].

The extraction method followed the study of Silva-Maia et al. [37]. The powder was mixed with distilled water (boiling, 100 °C) in a glass beaker. A sample-to-solvent ratio of 1:20 was applied. The mixture was allowed to rest for 30 min at room temperature with eventual mixing at 0, 15, and 30 min. The resulting infusion was filtered through a paper filter (0.2 mm) and stored at −20 °C. The residue was discarded. The extract was freeze-dried and quantified regarding the levels of monomeric anthocyanins [29]. The extract preparation and analysis were performed in triplicate.

Caco-2, HT29, and HT29-MTX cell lines were obtained from the European Collection of Authenticated Cell Cultures (ECACC, Salisbury, UK). They were grown in high-glucose DMEM, supplemented with 10% fetal bovine serum and 1% penicillin/streptomycin solution, and kept at 37°C with 5% CO_2_.

For the cell viability assay, human CRC cells, Caco-2, HT29, and HT29-MTX, were seeded at 10,000 cells per well (150 μL) in 96-well flat-bottom plates. The treatment was carried out after 72 h of seeding (cells have reached at least 70% confluence). For the treatment, the following concentrations (mg/mL) of freeze-dried extract were applied: 0, 0.025, 0.05, 0.1, 0.5, 1, and 2. The extracts were diluted in fresh media. After 24 or 48 h of treatment, to measure cell death/survival, the cells were submitted to the MTT protocol. Briefly, the wells were added with 50 μL serum-free media and 50 μL MTT solution, and incubated at 37 °C for 3 h. After incubation, the content was retrieved and 150 μL of MTT solvent was added to each well, followed by orbital shaking until homogenization. The absorbance was read at 590 nm. The values were subtracted from blank values and the absorbance readings of the treatments were compared to those of the control (concentration of 0 mg/mL) in order to compose the final percentage results, which were indicative of cell survival or cell death/toxicity. The half maximal inhibitory concentration (IC50) was calculated using the percentage values of cell death; the ED50plus v1.0 program was used. Three replicates were performed for each cell line tested.

For the colony formation assay, the cell line with the most effective anti-proliferative effect in the MTT assay was tested. Two assays were carried out with distinct seeding and extract concentrations. The cells were seeded at 500 or 1000 cells per well using 6- or 24-well flat-bottom plates, respectively. After 24 or 48 h, the wells were added with the extracts at concentrations of 0, 0.5, 1, and 2 mg/mL (6-well plate) or 0, 0.025, 0.05, 0.1, 0.25, 0.5, 0.75, 1, 1.5, and 2 mg/mL (24-well plate), respectively. The extracts were diluted in fresh media. Every three days, new media was provided to cells. After a total of 8 days, the crystal violet protocol was applied. For the crystal violet protocol, cells were washed twice with PBS and incubated with absolute methanol for 20 min. The content was retrieved and a 0.5% crystal violet solution was added to the wells for 40 min. After that, the crystal violet solution was retrieved and the wells were washed with PBS up to four times. The plates were left at room temperature to dry. Photos of the wells were taken in the GelCount^TM^ equipment (Oxford Optronix, Oxford, UK) for a visual comparison between the treatments. The assays were performed in triplicate.

### 2.6. Statistical Analysis

Data are represented by mean ± standard deviation (SD). Data were initially submitted to a normality test considering kurtosis and skewness values between −2 and 2 [38]. The composition differences between jaboticaba harvest periods (two samples) were analyzed by Student’s *t*-test (Welch’s correction, two-tailed). Data from the antiproliferative experiments were compared by One-way ANOVA followed by Tukey; the jaboticaba treatments (0.025–2 mg/mL) were compared to the control wells (concentration of 0 mg/mL). A *p* < 0.05 was considered to determine statistical difference, being represented in figures by the asterisk symbol (*).

## 3. Results and Discussion

### 3.1. Chemical Composition and Antioxidant Capacity

Sample 2 (August–October) showed a significantly higher (*p* < 0.05) moisture content after lyophilization when compared to sample 1 (May). Both samples went through the same freezing, thawing, and lyophilization procedures; therefore, differences in moisture after drying are probably an influence of jaboticaba’s collection periods. An aspect that may have exerted influence is that sample 2 comes from jaboticaba collected during three months, while sample 1 was collected over only one month. For example, the study of Bower and Papli [39] found variable moisture percentages (~60–80%) in the avocado fruit collected in different months (April–September). Also, Garçoa et al. [40] found moisture values varying greatly between jaboticaba’s developing moments, with differences between them reaching 14% within 34 days.

**Figure 1:**
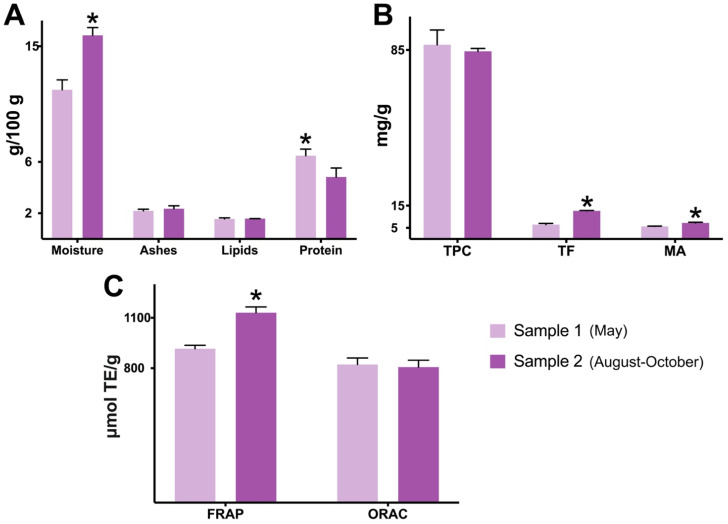
**Proximate and spectrophotometric composition differences between two samples of jaboticaba peel powder.** Except for moisture, all results are in dry weight of jaboticaba peel powder. Sample 1: jaboticaba collected in May; sample 2: jaboticaba collected in August–October. Data are represented by mean ± standard deviation (SD). Student’s *t*-test (Welch’s correction, two-tailed); the asterisk symbol (*) indicates statistical difference (*p* < 0.05) between samples 1 and 2. (**A**) Proximate composition. (**B**) Spectrophotometric composition. (**C**) Antioxidant capacity. Abbreviations: FRAP—ferric-reducing antioxidant power; MA—monomeric anthocyanins; ORAC—oxygen radical absorbance capacity; TE—Trolox equivalent; TF—total flavonoids; TPC—total phenolic content.

Despite having the highest moisture amount, sample 2 displayed a lower content of protein (4.82 ± 0.70) when compared with sample 1 (6.47 ± 0.51 g/100 g, dry weight) (*p* < 0.05) (Figure 1A). Other literature findings indicate that jaboticaba peel powders are usually low sources of protein, ranging between 3.77 and 7.31 g/100 g [22,36,41,42]. The powders’ contents in ashes and lipids were also relatively low and similar between the samples (Figure 1A). The total carbohydrate content for both jaboticaba powders was estimated at 89–92% (dry weight).

In terms of bioactive compounds, sample 2 showed significantly higher (*p* < 0.05) amounts of total flavonoids (92% richer; 12.62 ± 0.19 mg/g, dry weight) and monomeric anthocyanins (28% richer; 7.22 ± 0.28 mg/g, dry weight) when compared to sample 1 (*p* < 0.05) (Figure 1B). Overall, both jaboticaba samples were revealed as “high sources” of TPC, since the samples surpassed 50 mg/g (dry weight), based on the classification of Rufino et al. [43]. Despite sample 2 having more content in flavonoids, the levels of TPC were not significantly changed by jaboticaba’s distinct harvest seasons (Figure 1B). The lower content of flavonoids found in sample 1 was compensated by the richness in gallic acid, which explains the lack of difference between the samples’ TPC. Another possibility is that sample 1 has more interfering agents, such as reducing sugars and/or ascorbic acid, factors known to overestimate the analysis values [44].

Sample 2 also showed a higher antioxidant capacity by FRAP (*p* < 0.05), which is probably associated with its superior levels of flavonoids and anthocyanins. However, the ORAC method did not capture any significant difference, since sample 2 showed a similar value to sample 1 (Figure 1C). The ORAC values found by our study (806.17 ± 41.09 and 821.80 ± 38.61) were similar to the one of Inada et al. [6] (827 µmol Trolox equivalent/g), also using *M. jaboticaba* peel powder. Among jaboticaba’s various fractions (whole fruit, pulp, peel, seed, and depulping residue), the authors confirmed that the peel possesses the most promising composition, given its significantly elevated contents in TPC and anthocyanins, in addition to a higher antioxidant capacity measure by ORAC [6]. When the ORAC values of well-known berries, including blackberry (423), raspberry (161), and strawberry (356 µmol Trolox equivalent/g) [45], are compared with the ones of jaboticaba peel (>806 µmol Trolox equivalent/g), the latter stands out as being a more promising antioxidant crop.

**Figure 2:**
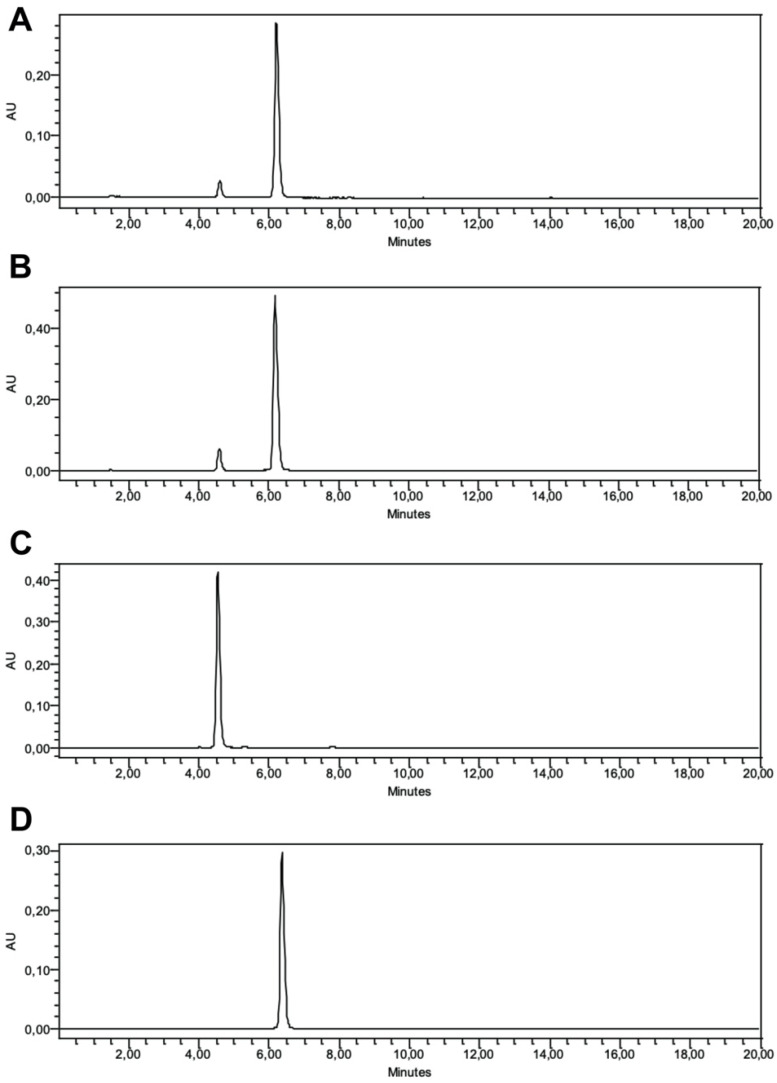
**Anthocyanin chromatograms of jaboticaba peel samples and standards.** The analyses were carried out in a high-performance liquid chromatography coupled with a diode array detector. (**A**) Jaboticaba peel, sample 1 (May). (**B**) Jaboticaba peel, sample 2 (August–October). (**C**) Delphinidin-3-*O*-glucoside standard. (**D**) Cyanidin-3-*O*-glucoside standard.

Following spectrophotometry, chromatography was performed for specific information on jaboticaba’s bioactive profile. The anthocyanin chromatograms revealed two major peaks for both samples 1 (Figure 2A) and 2 (Figure 2B). With the aid of purified standards, these were identified (from left to right) as D3G for the first peak (Figure 2C) and C3G for the second peak (Figure 2D). The peak for C3G was way more prominent than D3G, and sample 2 showed a higher AU reach for both C3G and D3G in comparison with sample 1. The P3G peak was not seen in any of the jaboticaba samples, indicating its absence in the fruit. The results found are in concordance with most HPLC investigations performed in jaboticaba peel powders, which indicate a clear dominance of C3G among total anthocyanins (about 80–90%) [46,47,48]. Unlike our study, a few other investigations have identified P3G in the peel, in addition to other less common anthocyanin derivatives from cyanidin, malvidin, pelargonidin, and petunidin compounds [49,50,51,52].

**Figure 3:**
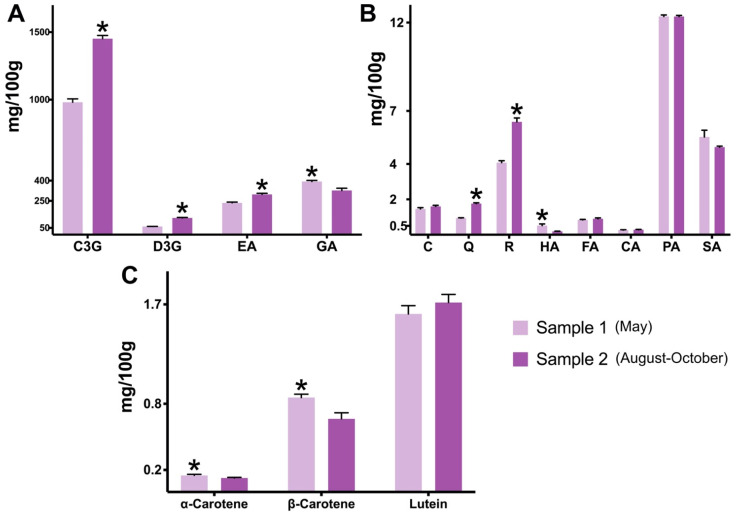
**Chromatographic composition differences between two samples of jaboticaba peel powder.** The analyses were carried out in a high-performance liquid chromatography coupled with a diode array detector. All results are in dry weight of jaboticaba peel powder. Sample 1: jaboticaba collected in May; sample 2: jaboticaba collected in August–October. Data are represented by mean ± standard deviation (SD). Student’s *t*-test (Welch’s correction, two-tailed); the asterisk symbol (*) indicates statistical difference (*p* < 0.05) between samples 1 and 2. (**A**) The composition of jaboticaba peel’s main polyphenols. The values for ellagic acid represent the sum of free and hydrolyzed fractions. The values for gallic acid represent the hydrolyzed fraction. (**B**) Other flavonoids and phenolic acids composition. The values for catechin, quercetin, rutin, and protocatechuic acid represent the sum of free and hydrolyzed fractions. The values for epicatechin, 4-hydroxybenzoic acid, ferulic acid, and *p*-coumaric acid represent the hydrolyzed fraction. The values for syringic acid represent the free fraction. (**C**) Carotenoids’ composition. Abbreviations: C3G—cyanidin-3-*O*-glucoside; D3G—delphinidin-3-*O*-glucoside; C—catechin; CA—*p*-coumaric acid; EA—ellagic acid; FA—ferulic acid; GA—gallic acid; HA—4-hydroxybenzoic acid; PA—protocatechuic acid; Q—quercetin; R—rutin; SA—syringic acid.

In general, the chromatographic analyses revealed that both powders are major sources (descending order) of C3G, gallic acid, ellagic acid, and D3G (Figure 3A), with fewer contents (<13 mg/100 g) of other flavonoids and phenolic acids (Figure 3B). The samples’ spectrophotometric differences in terms of flavonoids and anthocyanins were also seen in chromatography. When compared to sample 1, sample 2 showed a 48% higher content in C3G (1451 ± 23.29 mg/100 g) and 105% higher in D3G (123.88 ± 3.58 mg/100 g) (*p* < 0.05) (Figure 3A), in addition to significantly elevated contents of free and hydrolyzed fractions of quercetin and rutin (*p* < 0.05) (Figure 3B). The C3G amount in sample 2 was similar to or higher than the ones found by the studies of Resende et al. [47,53] (*Plinia* spp., 352–1008), Leite-Legatti et al. [22] (*M. jaboticaba,* 1514), and Quatrin et al. [51] (*M. trunciflora*, 1039, and *M. jaboticaba*, 1636 mg/100 g), but lower than those reported by Leite et al. [54] (*M. jaboticaba*, 1966) and Plaza et al. [48] (*M. jaboticaba*, 2866 mg/100 g). In general, most studies using jaboticaba peel, including ours, have employed acetic or formic acid to acidify the extraction solutions and obtain high yields of anthocyanins for HPLC analyses. These weakly acid solvents are known to provide a favorable extraction and avoid the hydrolysis of not only common anthocyanins but also acylated and 3,5-diglucosides, therefore increasing their specific and total content [55].

Gallic and ellagic acids were by far the most abundant phenolic acids found in the jaboticaba powders, followed by (in descending order) protocatechuic acid and syringic acid (Figure 3B). While sample 2 showed the highest levels of total ellagic acid (free and hydrolyzed fractions) (*p* < 0.05), sample 1 displayed the most significant contents in hydrolyzed phenolic acids, these being gallic acid and 4-hydroxybenzoic acid (*p* < 0.05) (Figure 3B). Especially, sample 1 demonstrating a significantly higher (*p* < 0.05) content in gallic acid (393.66 ± 7.84 mg/100 g), by 20%, should be highlighted, as this phenolic acid has been considered a possible candidate for the treatment of gastrointestinal diseases, given its modulatory interaction with the gut microbiota and immune response [56]. Recently, jaboticaba peel’s richness in not only anthocyanins but also phenolic acids, including gallic acid, has been associated with the inhibition of colorectal carcinoma in mice [14]. The levels of catechin, ferulic acid, *p*-coumaric acid, protocatechuic acid, and syringic acid did not differ statistically between the samples (Figure 3B); and epicatechin was not identified in either jaboticaba powder.

The characterization of carotenoids was performed to facilitate an extended comparison between the harvest seasons. Jaboticaba’s carotenoid composition, especially, has been poorly documented in the literature. The powders’ chromatographic levels of α-carotene, β-carotene, and lutein, when combined (2.5–2.7), were slightly lower than the ones shown by the total carotenoids analysis (3.07 ± 0.12 and 3.21 ± 0.10 mg/100 g). The literature has reported a total carotenoid content varying between 1 and 5 mg/100 g for jaboticaba peel powders [57,58,59], which is in accordance with our study. No significant differences in total carotenoid content were found between the samples. Regarding specific carotenoids, lutein was the most common compound found in the jaboticaba powders (Figure 3C), representing between 60 and 70% of the total carotenoid content shown by chromatography. Similarly, Biazotto et al. [60] showed that lutein is the most common carotenoid found in the pulp, along with the peel of *P. cauliflora*. On the other side, Inada et al. [6] only found β-carotene among the carotenoids profile of *M. jaboticaba*’s whole fruit, with a content (0.87) similar to our study (0.85 ± 0.03 mg/100 g, sample 1). β-cryptoxanthin was not identified in the jaboticaba powders of the present study. When the harvesting periods were compared, sample 1 was revealed as a better source of α-carotene and β-carotene when compared to sample 2 (*p* < 0.05) (Figure 3C). However, overall, jaboticaba’s carotenoid content is less relevant than other compounds, such as flavonoids and phenolic acids.

The present study is a rare report on the composition differences between two jaboticaba harvesting seasons. Additionally, it highlights an uncommon collection period (May) for the fruit. Previous investigations have shown that the levels of bioactive compounds can change depending on the physiological and maturation developments of the fruit, but always analyzing within the year’s second semester or jaboticaba peak season. These studies have been conducted in the various fractions (peel, pulp, and seed) of *M. cauliflora,* variety ‘Pingo de mel’, and showed that, to obtain the highest levels of anthocyanins, essential oils, and vitamin C, the collection should be carried out 30 to 37 days after flowering [61,62,63]. More investigations with jaboticaba collected in unusual seasons may reveal the fruit’s other interesting characteristics and chemical attributes. In addition, it is also important that further studies collect and analyze samples from different years. In our study, the fruits from both peak season and off-season were obtained in the same year, limiting a more accurate comparison in the long-term and a better comparison with the literature.

All numeric values from the composition and antioxidant capacity analyses can be seen in the Supplementary Materials.

### 3.2. Anti-Cancer Activity

For the anti-cancer analyses, the treatment of choice was the freeze-dried aqueous peel extract from the jaboticaba collected in August–October (peak season, sample 2), as this sample showed the most content of anthocyanins from both spectrophotometric and chromatographic analyses. When analyzed for monomeric anthocyanins, the extract showed a value of 8.13 ± 1.32 mg/g. Additionally, a study of our research group [64], using the same extraction method, as well as the same fruit fraction, species, and supplier, identified and quantified the following compounds as the main polyphenols (in descending order) in the aqueous sample: C3G, gallic acid, rutin, kaempferol-3-*O*-glucoside, and D3G. C3G appears to be a remarkable and sometimes dominant compound in jaboticaba extracts, independent of the extraction method.

**Figure 4:**
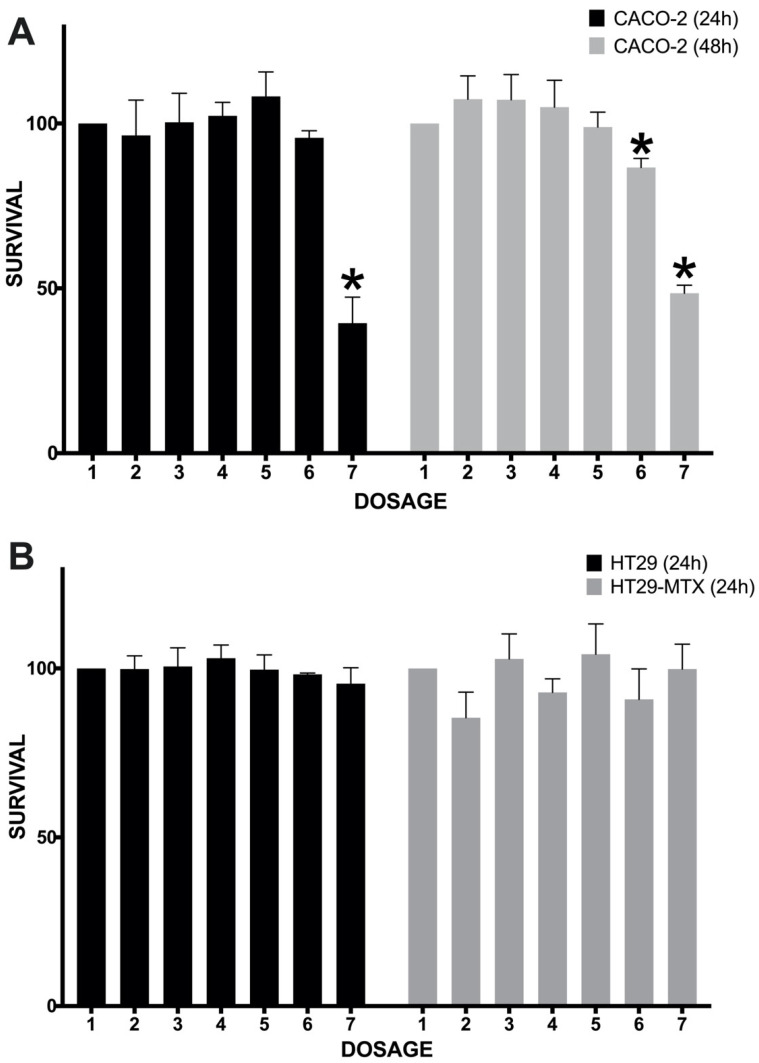
**The anti-proliferative activity of jaboticaba peel extract (August–October sample) on colorectal cancer cell lines.** Seeding: 10,000 cells per well. Extract concentrations: 1/control (0), 2 (0.025), 3 (0.05), 4 (0.1), 5 (0.5), 6 (1), and 7 (2 mg/mL). Cell death/survival was measured by the MTT assay. Data are represented by mean ± standard deviation (SD). One-way ANOVA followed by Tukey; the asterisk symbol (*) indicates statistical difference (*p* < 0.05) (the jaboticaba treatments were compared to the control concentration). (**A**) Survival of Caco-2 cells after exposure for 24 or 48 h of jaboticaba peel extract. (**B**) The survival of HT29 and HT29-MTX cells after exposure for 24 h of jaboticaba peel extract.

The extract’s highest concentrations, 1 and 2 mg/mL, were capable of significantly reducing the survival of Caco-2 cells after 48 h and 24/48 h of extract exposure, respectively (Figure 4A). Especially, the 2 mg/mL concentration was powerful enough to kill on average about 50–60% of all cells. The IC50 values were calculated as 1.32 ± 0.06 and 1.21 ± 0.07 mg/mL for the treatment times of 24 and 48 h, respectively. The 48 h treatment was slightly more effective, as two concentrations reduced cancer cell proliferation (Figure 4A). When compared to other investigations, the IC50 found by our study may be considered higher than expected, although still effective for an extract prepared without toxic and non-green solvents. Studies using other jaboticaba-based extracts have shown the following best IC50 values against CRC cell lines: 0.14 (HT29) [22], 0.34 (HCT116) [13], 0.45 (Caco-2) [18], 0.76 (HT29) [15], and 37.52 mg/mL (HT29 cells) [16].

Previously, the cell viability of Caco-2 cells after treatment with a jaboticaba-based product has only been investigated once. Fidelis et al. [18] treated Caco-2 cells for 48 h with an aqueous/propanone-based jaboticaba (*M. jaboticaba*) seed extract, finding significative antiproliferative effects starting at a concentration of 0.5 mg/mL. In addition, investigations have confirmed the ability of anthocyanin-rich extracts to reduce the proliferation of Caco-2 cells. These include grape byproducts (seed, pomace) [65], black raspberry [66], and other Brazilian fruits, such as açaí (*Euterpe oleracea*) [67] and tucum-do-Cerrado (*Bactris setosa*) [68]. Anthocyanins’ mechanisms on CRC cells are still being understood, with experimental articles also indicating the participation of a few extrinsic mediators. C3G, for example, which is the main anthocyanin found in jaboticaba, has been linked with the blockage of programmed death-ligand 1 in HCT-116 cells [69], a scenario said to reduce the protein’s bond with programmed death-1 receptor and promote the reactivation and anti-cancer action of cytotoxic T lymphocytes [70]. Another report suggests that C3G may also act by downregulating the epidermal growth factor receptor (EGFR) [71], therefore promoting increased apoptosis and reduced angiogenesis mechanisms [72]. The anti-proliferative effects found by the present study are more likely to be associated with a blockage of EGFR, as we did not culture CRC cells with lymphocytes.

The present study also tested the effects of jaboticaba against other CRC cell lines. In general, HT-29 has been the most tested cell line on investigations with jaboticaba extracts. Previous studies have found anti-proliferative effects against HT29 by using aqueous or dichloromethane/ethanol extracts from jaboticaba peel [22,46], as well as colon-fermented samples [15]. However, our results with HT29-based cell lines did not come out as promising. The treatment with the peel’s aqueous extract had no inhibitory effects on HT29 or HT29-MTX cell lines (Figure 4B). Similarly, Nascimento et al. [13] did not manage to abrogate HT29 cells when testing a pectin-rich extract from jaboticaba (*M. cauliflora*) whole fruit. Such inefficacy is probably related to one or more of the following aspects of our study: (a) the concentrations applied may be too low to achieve proper inhibition results; (b) an aqueous extract usually has a lower content in total polyphenols and anthocyanins when compared with other commonly used solvent-based extracts; and (c) more treatment time may be necessary to achieve better responses. Caco-2 and HT29 cells’ characteristics and metabolism differences could also help explain such responses. When uncovering the gene expression profile between various CRC cell lines, Bourgine et al. [73] found the lowest correlation coefficients for Caco-2 and HT29 cells. While Caco-2 cells present a higher homology to enterocytes in the intestinal epithelium, in addition to most receptors, transporters, and drug-metabolizing enzymes, HT29 cells express more mucin and linked factors [74,75]. In our study, only Caco-2 cells showed sensitivity towards the aqueous extract of jaboticaba peel.

**Figure 5:**
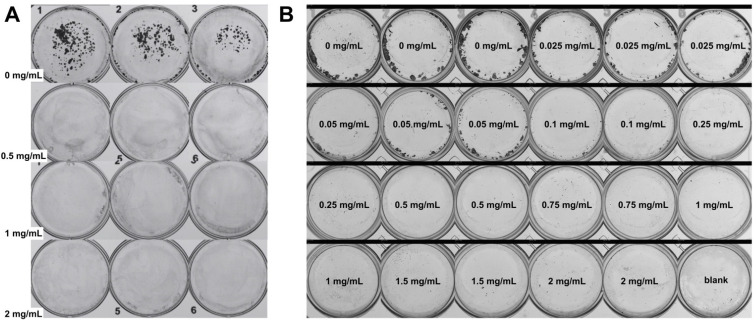
**The anti-colony-forming activity of jaboticaba peel extract (August–October sample) on Caco-2 cells.** The crystal violet protocol was applied to observe cell colony formation. (**A**) First assay. Seeding: 500 cells per well. Extract concentrations: 0, 0.5, 1, and 2 mg/mL (6-well plate). (**B**) Second assay. Seeding: 1000 cells per well. Extract concentrations: 0, 0.025, 0.05, 0.1, 0.25, 0.5, 0.75, 1, 1.5, and 2 mg/mL.

Considering the promising anti-proliferative effects of jaboticaba against Caco-2 cells, we used this cell line to identify if the extracts could inhibit cell colony formation. As a result, in the first assay, colonies were formed only in the first three control wells (horizontal direction), where no treatment was applied. The 0.5, 1, and 2 mg/mL concentrations were able to effectively inhibit colony formation (Figure 5A). In the second assay, on which 10 concentrations were tested, it was possible to notice that increasing the concentration lowered the number of colonies. Starting at concentration 0.1 mg/mL, almost no colonies were formed (Figure 5B). When compared to the MTT assay, the colony formation scheme showed more sensitivity towards the action of jaboticaba peel, as low concentrations were revealed to be efficient. Technically, the longer treatment time and fewer cells seeded could have contributed to more significant results in the colony formation assay. In terms of the biological mechanism, however, the results may be explained by the extract’s capacity to positively alter Caco-2’s genes and markers associated with intestinal stem cells, which are considered a hallmark of CRC initiation [76]. Recently, May et al. [76] showed that anthocyanin-rich black raspberry holds potential for CRC prevention, as it effectively reduces cancer cell stemness and linked markers of early tumorigenesis, consequently extending lifespan and decreasing tumor development. Natural plant extracts could be considered a strategy in progress to block CRC initiation, especially for those at high risk of obtaining the disease.

A recent investigation by Silva-Maia et al. [77] showed that *M. jaboticaba* has anti-colony-forming properties against breast cancer cells. The authors tested the peel’s hydroethanolic- and ethyl acetate-based extracts and found reduced numbers of colonies for MDA-MB-231 cells when applied at the concentrations 0.01–0.025 and 0.1 mg/mL, respectively [77]. According to our search, the present study is the first report on jaboticaba’s anti-colony effects in a CRC cell line.

It is important to highlight the limitations of the in vitro study. First, we did not perform comparisons between distinct extraction methods or solvents; therefore, it is not possible to confirm that an easy-to-prepare aqueous extract would be more or less effective (or the best option) against CRC cell lines. Additionally, we did not perform comparisons with other fruit-like products or drugs, limiting our analogies to the results of the literature. Finally, our study did not investigate the metabolism and cellular mechanisms of jaboticaba compounds in vitro, a piece of information that could help explain with more certainty the antiproliferative and anti-colony-forming results found. It would be important for future studies to analyze the expression or concentration of markers related to inflammation, oxidative stress, and apoptosis, including cytokines, caspases, and binding proteins.

## 4. Conclusions

Jaboticaba peel’s chemical composition can be considerably changed depending on the fruit’s harvesting season. If the goal is obtaining the highest bioactive-enriched product, the peel from the fruit collected in the peak season (August–October) presents the most potential, given its superior levels of anthocyanins. Despite that, the May sample must not disregarded, as it still contains high amounts of polyphenols, especially gallic acid, and therefore, holds pro-health potential as well. In that respect, our study also showed that an eco-friendly aqueous extract from jaboticaba peel has anti-proliferative and anti-colony-forming effects on Caco-2 cells. The effects found strengthen the current knowledge regarding the fruit’s applicability against intestinal malignancies. Future pre-clinical studies should promote better insight into the molecular mechanisms by which jaboticaba peel acts to block the growth of CRC cells. In addition, clinical trials could be addressed in the same way as studies with other anthocyanin-rich fruits (bilberry, black raspberry) have been conducted in the last decade.

## Data Availability

The raw data supporting the conclusions of this article will be made available by the authors on request.

## Supplementary Materials

The following supporting information can be downloaded at: https://www.mdpi.com/article/10.3390/plants13202907/s1.

## Abbreviations

C3G	cyanidin-3-*O*-glucoside
CRC	colorectal cancer
D3G	delphinidin-3-*O*-glucoside
DAD	diode array detector
DMEM	Dulbecco’s Modified Eagle Medium
EGFR	epidermal growth factor receptor
FRAP	ferric-reducing antioxidant power
HPLC	high-performance liquid chromatography
IC50	half maximal inhibitory concentration
ORAC	oxygen radical absorbance capacity
P3G	pelargonidin-3-*O*-glucoside
PBS	phosphate-buffered saline
TPC	total phenolic content
