# Supplementary material for: Myrciaria jaboticaba Fruit Peel: Bioactive Composition as Determined by Distinct Harvest Seasons and In Vitro Anti-Cancer Activity
Source: Plants (Basel). 2024 Oct 17;13(20):2907. doi: 10.3390/plants13202907 (PMC11510877; doi:10.3390/plants13202907)
Supplement: Supplementary file 1 [file plants-13-02907-s001.zip › plants-3196270-supplementary.pdf]

SUPPLEMENTARY MATERIAL

Proximate, spectrophotometric, and chromatographic characterization of jaboticaba Sabará (*Myrciaria jaboticaba* (Vell.) O. Berg) peel powders

**Analysis**	**May sample**	**August-October sample**
**PROXIMATE** (g/100 g)		
Moisture	11.60 ± 0.77	15.85 ± 0.60*
Ashes	2.18 ± 0.13	2.35 ± 0.22
Lipids	1.55 ± 0.08	1.58 ± 0.001
Protein	6.47 ± 0.51*	4.82 ± 0.70
**SPECTROPHOTOMETRIC**		
Total phenolic content (mg GAE/g)	87.34 ± 6.64	84.36 ± 1.31
Total flavonoids (mg quercetin equivalent/g)	6.47 ± 0.51	12.62 ± 0.19*
Monomeric anthocyanins (mg C3G/g)	5.64 ± 0.12	7.22 ± 0.28*
Total carotenoids (mg/100 g)	3.21 ± 0.10	3.07 ± 0.12
FRAP (µmol Trolox equivalent/g)	915.90 ± 20.18	1130.58 ± 34.12*
ORAC (µmol Trolox equivalent/g)	821.8 ± 38.61	806.17 ± 41.09
**CHROMATOGRAPHIC (HPLC-DAD)** (mg/100g)		
**Flavonoids**		
Cyanidin-3-*O*-glucoside	980.68 ± 25.34	1451.80 ± 23.39*
Delphinidin-3-*O*-glucoside	60.26 ± 0.77	123.88 ± 3.58*
Pelargonidin-3-*O*-glucoside	ND	ND
Catechin+	1.46 ± 0.07	1.60 ± 0.06
Epicatechin#	ND	ND
Quercetin+	0.93 ± 0.02	1.76 ± 0.04*

Rutin+	4.07 ± 0.11	6.38 ± 0.21*
**Phenolic acids**		
4-Hydroxybenzoic acid#	0.51 ± 0.09*	0.19 ± 0.01
Ellagic acid+	235.25 ± 5.89	298.24 ± 7.69*
Ferulic acid#	0.83 ± 0.03	0.90 ± 0.05
Gallic acid#	393.66 ± 7.84*	327.44 ± 16.51
p-Coumaric acid#	0.26 ± 0.02	0.28 ± 0.02
Protocatechuic acid+	12.35 ± 0.07	12.34 ± 0.06
Syringic acid**&**	5.53 ± 0.38	4.95 ± 0.05
**Carotenoids**		
α-Carotene	0.15 ± 0.01*	0.12 ± 0.006
β-Carotene	0.85 ± 0.03*	0.66 ± 0.05
β-Cryptoxanthin	ND	ND
Lutein	1.61 ± 0.07	1.71 ± 0.07

Results are expressed in mean ± standard deviation (SD). Except for moisture, all results are in dry weight of jaboticaba peel powder. Student's t-test was performed between the samples; the asterisk symbol (*) indicates statistical difference ($p<0.05$). +Sum of free and hydrolyzed fractions. #Hydrolyzed fraction. **&**Free fraction**.** Abbreviations: FRAP: ferric reducing antioxidant power, GAE: gallic acid equivalent, HPLC-DAD: high-performance liquid chromatography coupled with a diode array detector, ND: not detectable, ORAC: oxygen radical absorbance capacity.
